# Identification of BCL11A, NTN5, and OGN as Diagnosis Biomarker of Papillary Renal Cell Carcinomas by Bioinformatic Analysis

**DOI:** 10.15586/jkc.v12i1.366

**Published:** 2025-02-28

**Authors:** Zahra Haghshenas, Sina Fathi, Alireza Ahmadzadeh, Elham Nazari

**Affiliations:** 1 Proteomics Research Center, Shahid Beheshti University of Medical Sciences, Tehran, Iran;; 2 Department of Health Information Technology and Management, School of Allied Medical Sciences, Shahid Beheshti University of Medical Sciences, Tehran, Iran;; 3 Departement of Laboratory Sciences, School of Allied Medical Sciences, Faculty of Paramedical Sciences, Shahid Beheshti University of Medical Sciences

**Keywords:** BCL11A, biomarker, diagnosis, machine learning, NTN5, OGN, PRCC

## Abstract

The prevalence of papillary renal cell carcinomas (PRCCs) is estimated to be between 10% and 15%. At present, there is no effective therapeutic approach available for patients with advanced PRCCs. The molecular biomarkers associated with PRCC diagnoses have been rarely studied compared to renal clear cell carcinomas; therefore, the necessity for the identification of novel molecular biomarkers to aid in the early identification of this disease. Bioinformatics and artificial intelligence technologies have become increasingly important in the search for diagnostic biomarkers for early cancer detection. In this study, three genes—BCL11A, NTN5, and OGN—were identified as diagnostic biomarkers using the Cancer Genome Atlas (TCGA) database and deep learning techniques. To identify the differential expression genes (DEGs), ribonucleic acid (RNA) expression profiles of PRCC patients were analyzed using a machine learning approach. A number of molecular pathways and coexpressions of DEGs have been analyzed and a correlation between DEGs and clinical data has been determined. Diagnostic markers were then determined via machine learning analysis. The 10 genes selected with the highest variable importance value (more than 0.9) were further investigated, with six upregulated (BCL11A, NTN5, SEL1L3, SKA3, TAPBP, SEMA6A) and four downregulated (OGN, ADCY4, SMOC2, CCL23). A combined receiver operating characteristic (ROC) curve analysis revealed that the BCL11A-NTN5-OGN genes, which have specificity and sensitivity values of 0.968 and 0.901, respectively, can be used as a diagnostic biomarker for PRCC. In general, the genes introduced in this study may be used as diagnostic biomarkers for the early diagnosis of PRCC, thus providing the possibility of early treatment and preventing the progression of the disease.

## Introduction

The incidence of kidney cancer is on the rise worldwide, making it the 13th most common malignancy (1, 2). In 2018, GLOBOCAN data indicated that 403,000 people were diagnosed with kidney cancer every year, accounting for 2.2% of all cancer diagnoses. The number of cases diagnosed in men was 254,500 and those in women was 148,800 (3). The renal cell carcinoma (RCC) is a heterogeneous disease that can be classified into a range of subtypes based on their histological characteristics, including clear cell (ccRCC), papillary (PRCC), chromophobe (chRCC), collecting duct (ccRCC), and unclassified (4). It is estimated that 10–15% of RCCs are papillary renal cell carcinomas (PRCCs), the second most common subtype following clear cell renal cell carcinoma. PRCCs can be classified histologically into two types: type 1 and type 2. PRCCs of type 2 are heterogeneous groups that can be further subdivided based on genetic and molecular characteristics (5). Presently, patients with advanced PRCC do not have access to an effective therapeutic approach (6). In recent years, a number of markers have been discovered that can predict the therapeutic effect and the outcome of renal clear cell carcinomas, including mutations in VHL, VEGF, CAIX, and HIF1a/2a (7). Despite this, a few studies have been conducted on the molecular biomarkers associated with PRCC to predict its curative effect (8). Consequently, it is imperative to identify novel molecular biomarkers which will aid in early diagnosis, provide insight into the pathology of the disease, and allow the development of effective therapeutic strategies.

The analysis of whole-genome expression (transcriptomic) provides early cancer detection, diagnostics, clinical outcomes, and the potential for disease dissemination (9). It is now possible to obtain large amounts of cancer data from the medical research community because of the introduction of new technologies in medicine. A significant challenge for researchers is to be able to predict a cancer outcome accurately. Therefore, machine learning methods, a subfield of artificial intelligence that provides computers with the ability to learn without having to be explicitly programmed, have become an increasingly popular tool for medical researchers. By applying these techniques, patterns and relationships can be discovered and identified from complex datasets; they are also capable of predicting future outcomes of a given type of cancer (10–14). As a result, these techniques have become increasingly popular and various biomarkers have been identified for the diagnosis, prognosis, and treatment of a wide range of cancers, including breast, prostate, pancreatic, and colorectal cancers in recent years (15–18). TCGA (The Cancer Genome Atlas), an integrated collection of clinical information and gene sequencing data, allows systematic analysis of the molecular mechanisms underlying clinical features associated with cancers. It contributes to the improved diagnostic methods, and ultimately improves the survival prognosis of cancer patients by assessing the pathological stage, histological type, tumor grade, diagnosis, and prognosis of the disease (19-22). This study used TCGA database for gene expression proofing and machine learning to identify the differential expression genes (DEGs) of PRCC tumors. A machine learning-based algorithm was also used to identify molecular pathways, co-expressions of DEGs, and diagnostic markers associated with the disease.

## Material and Methods

### The collection of data

A total of 536 samples from the TCGA dataset, including RNA-seq (ribonucleic acid sequencing) data for kidney renal clear cell carcinoma (KIRC) patients and clinical features, such as sex, tumor stage, TNM (T: size of the tumor and spread of cancer into nearby tissue; N: spread of cancer to nearby lymph nodes; M: metastasis) classification, and survival profile, were obtained (http://tcga-data.nci.nih.gov/tcga/). For the following steps of RNA sequencing, 530 tumor tissues were collected and 6 normal tissues were selected from the KIRC samples.

### Identification of DEGs by preprocessing data

The identification of novel genes was performed using machine learning methods. As normalization and filtering are essential steps of data analysis, they were evaluated as preprocessing steps prior to applying machine learning to RNA data. Using R programming, duplicate genes and samples were omitted from the dataset as part of the filtering process. Then, 20,531 genes were normalized by using Limma and Edge R packages. DEGs were screened according to the particular criteria, which included the log fold change (logFC) ≥ 1.5 and P-value <.0.05. R software (version 4.01) was used to perform all the analysis as well as create the plots.

### Identification of predictive markers

Our research utilized deep learning (DL) to predict DEGs as important markers in PRCC through a bioinformatic analysis. The DL subgroup of machine learning focused on predicting outcomes with multilayered neural network algorithms derived from the neurological architecture of the human brain. With DL, neural network architecture allows models to scale exponentially with the increasing quantity and complexity of data as opposed to other ML methods such as logistic regression. Hence, DL is widely used to solve complex computational problems, such as the classification of large-scale images, the processing of natural language, and the recognition and translation of speech (23).

In order to implement machine learning, Python 3.7 was used. Python packages including Pandas, NumPy, Matplotlib, and Scikit-learn were applied. Based on the training data, models were optimized and independently evaluated. In step 5, a ratio of 40/60 to 95/5 was compared with a ratio of 70/30 to determine if methods of machine learning were approved. Area under the curve (AUC), accuracy, F1 score, R2 score, and confusion matrix were used to measure the performance of methods for identifying important genes.

In machine learning, accuracy is a metric for evaluating the degree to which the true positives and true negatives of machine learning classification are close to their true values. It is a method of categorizing imbalanced data into false positives and false negatives based on the degree of closeness between the measurement and its true value. AUC curve is a metric that determines whether a class can be correctly classified, and the area under the receiver operating characteristic (ROC) curve is represented by the AUC. The ROC curve is commonly used to assess predictive models’ discriminative abilities. The confusion matrix summarizes four types of classification (TN [True Negative], TP [True Positive], FN [False Negative], and FP [False Positive]) and defines the algorithm’s purpose. Performance models can be assessed using R2 score or coefficient of determination primarily in relation to feature selection (24–26).

### Functional and pathway enrichment

An analysis of functional enrichment and the identification of critical pathways as they relate to the DEGs signature were annotated and visualized with the clusterProfiler package in R with a p-adjusted < 0.05 using cluster correlation coefficients.

### Clinical data and DEGs correlation

In order to investigate if DEGs were correlated with clinical data such as age, tumor size, lymph node involvement, distant metastasis, and stage, 55 DEGs were analyzed using correlation matrixes and Spearman correlations in the R program using the ggcorrplot package and cor function in conjunction with the R program.

### Combine ROC curve

We assessed the diagnostic efficacy and developed diagnostic models using a generalized linear model and ROC curve analysis. For the assessment of the discrimination of individual or combined biomarkers, sensitivity, specificity, cut-off value, positive prediction, negative prediction, and area under the ROC curve were assessed. The entire procedure was implemented in R using the package combinoROC.

### Validation of gene expression biomarkers

Through the use of Global Data Assembly Centers (GDAC) (https://gdac.broadinstitute.org/) and Gene Expression Omnibus (GEO) datasets (GSE2748, GSE7023, GSE48352, GSE15641, and GSE26574), the expression levels of candidate genes in PRCC patients were examined. It was obtained from this web tool that the validation dataset, consisting of data from KIRC patients, was preprocessed.

## Results

### Patient demographics

The clinical data are shown in Table 1. Our population consisted of 346 males and 190 females, the mean age was 60.62 years, and were of three races: white, black, and Asian. Among the examined patients, 374 are alive and 162 died. About 39% of patients had advanced PRCC stage and the percentage of metastasis and lymph node involvement in patients was 14.7% and 3.2%, respectively.

**Table 1:** The Clinicopathological Characteristics Of Prcc Patients.

Clinicopathological Variables	No. of patients (%)/mean ±SD
Patients	536
Mean age (years, mean ±SD)	60.62 ±12.15
Sex	
Male	346 (64.6)
Female	190 (53)
Race	
Asian	8 (1.5)
White	466 (86.9)
Black	55 (10.3)
Missing data	7 (1.3)
Ethnicity	
Not Hispanic or Latino	358 (66.8)
Hispanic or Latino	26 (4.9)
Missing data	152 (28.4)
Vital status	
Dead	162 (30.2)
Alive	374 (69.8)
Stage	
0	2 (0.4)
1	268 (50)
2	57 (10.6)
3	125 (23.3)
4	84 (15.7)
Depth of tumor invasion (T)	
T1	274 (51.1)
T2	69 (12.9)
T3	182 (34)
T4	11 (2.1)
Lymph node involvement (N)	
No	240 (44.8)
Yes	17 (3.2)
Missing data	279 (52.1)
Metastasis (M)	
No	426 (79.5)
Yes	79 (14.7)
Missing data	31 (5.8)

### Identification of DEGs

**Figure 1:**
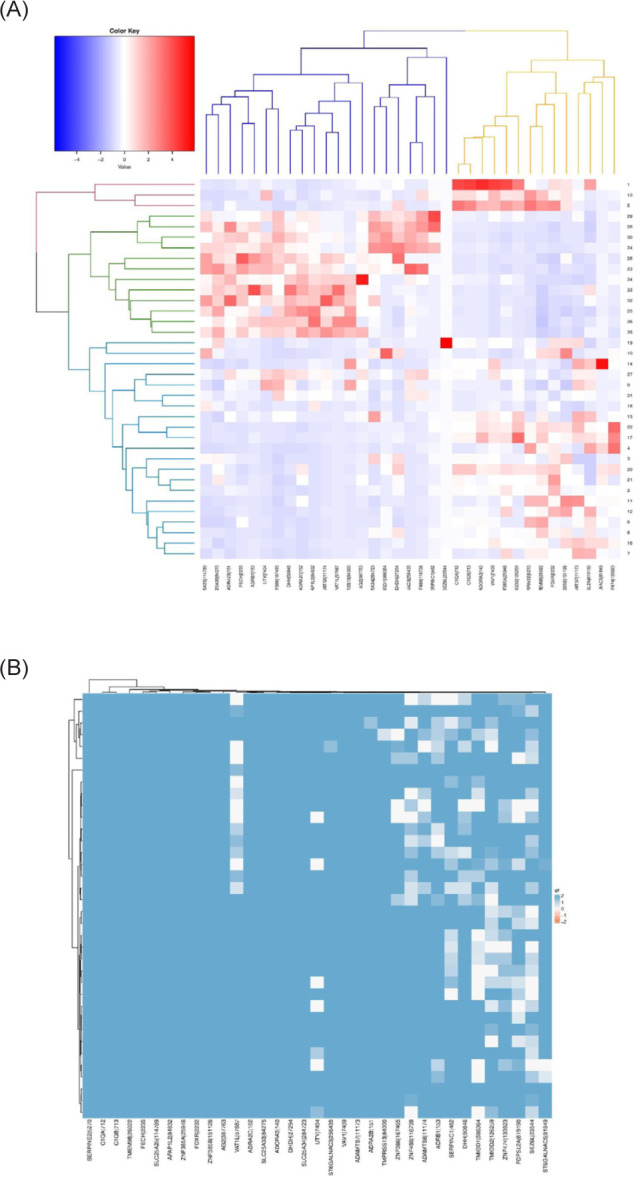
Heatmap of differential expression genes in PRCC patients was drawn by R software.

**Figure 2:**
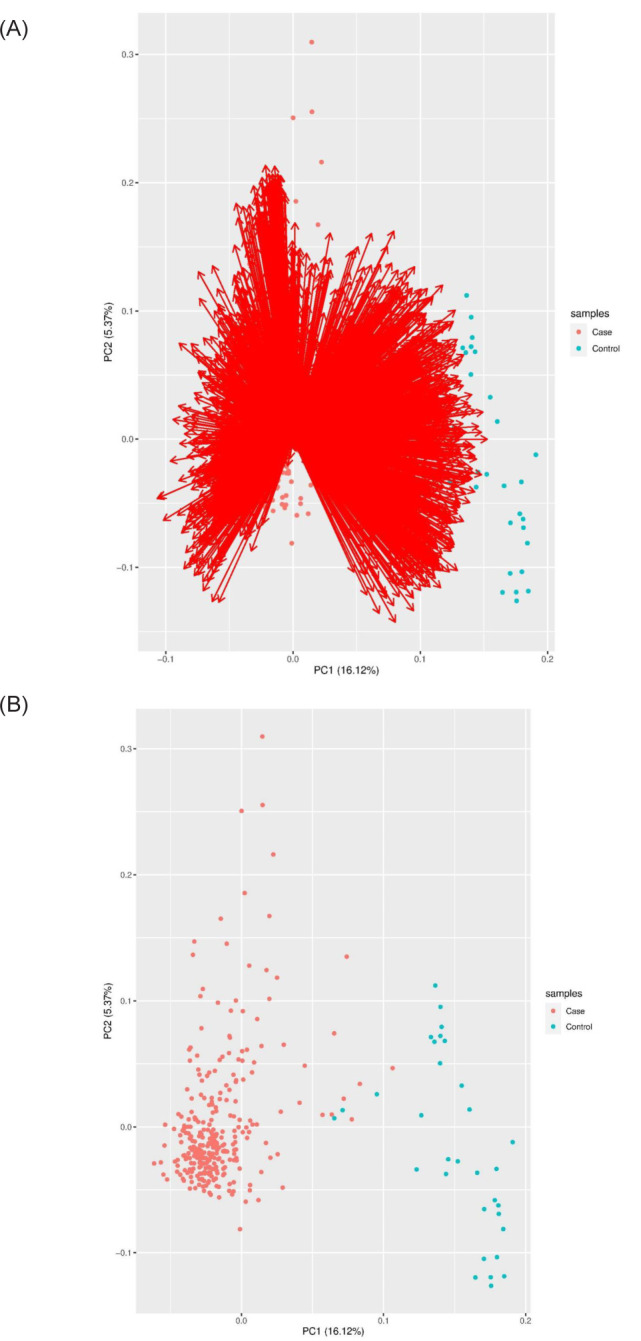
PCA of differential expression genes in PRCC patients.

The data were downloaded from TCGA comprised of 536 patients. After filtering and normalization, from 20,531 genes, we reached 3,229 DEGs (tumor vs. normal) that had the logFC ≥ 1.5 and P-value < 0.05, and a representation of genes can be seen in a heat map (Figure 1) and principal component analysis (PCA) (Figure 2).

### Identification of predictive markers

The key genes were analyzed by machine learning algorithm and DL, with the performance listed in Table 2. In general, 10 genes that had the highest variable importance (more than 0.9) were selected for further studies. Among them, six genes upregulated (BCL11A, NTN5, SEL1L3, SKA3, TAPBP, SEMA6A) and four genes downregulated (OGN, ADCY4, SMOC2, CCL23) (Table 3).

**Table 2:** Deep learning performance.

MSE	RMSE	R^2	AUC	Pr_AUC	Accuracy
1.1814987E-4	0.010869676	0.99867266	1.0	1.0	97.77%

**Table 3:** The top DEGs of TCGA were ranked by deep learning

Genes	Expression	Variable Importance
*BCL11A*	Up	1.000000
*NTN5*	Up	0.943672
*OGN*	Down	0.937124
*ADCY4*	Down	0.923404
*SMOC2*	Down	0.916624
*SEL1L3*	Up	0.916577
*CCL23*	Down	0.915636
*SKA3*	Up	0.911442
*TAPBP*	Up	0.911096
*SEMA6A*	Up	0.910605

### Functional and pathway enrichment

**Figure 3:**
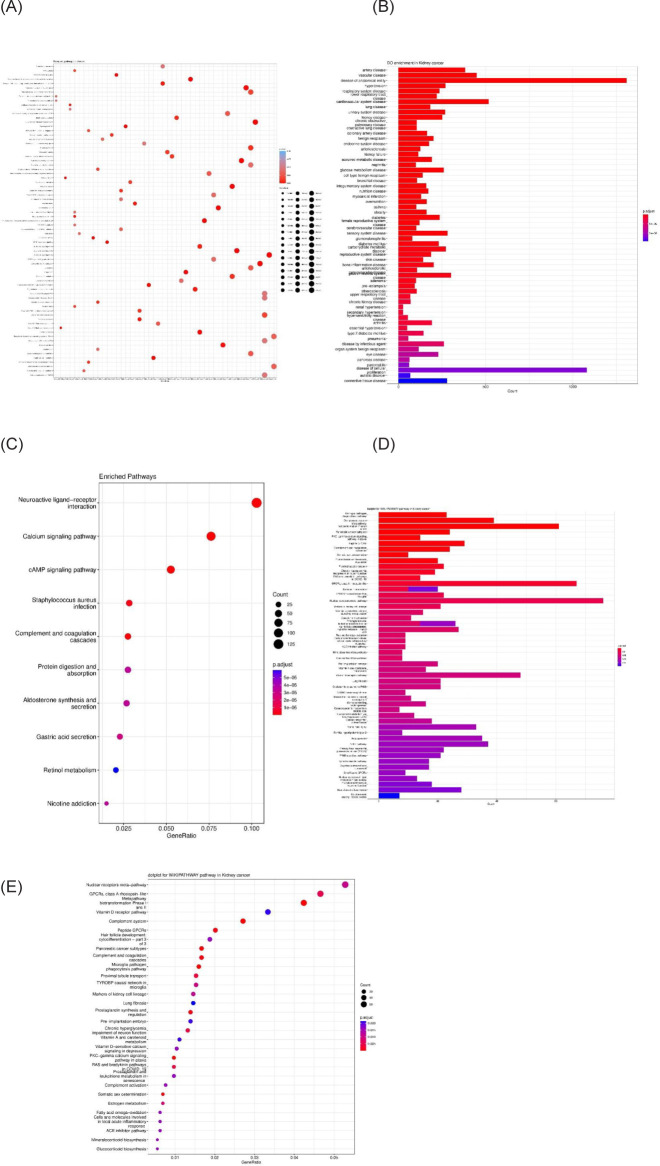
(A) Reactome pathway enrichment, (B) DO enrichment in kidney cancer, (C) Enriched pathways, (D) Barplot for WIKIPATHWAY pathway in kidney cancer, (E) Dotplot for WIKIPATHWAY pathway in kidney cancer. The P-value is less than 0.05 and is shown by the color.

Based on the R software, a total of key genes were enriched for their gene ontology and KEGG pathway analysis. The connection of genes in key and different pathways was identified, including calcium signaling pathway, cAMP signaling pathway, complement and coagulation cascades, protein digestion and absorption, aldosterone synthesis and secretion, vitamin D receptor pathway, signaling by GPCR, and more. In addition to PRCC, these genes also play a role in other diseases such as artery, vascular, cardiovascular system, urinary system, kidney, and benign neoplasm (Figure 3).

### Clinical data and DEGs correlation

**Figure 4:**
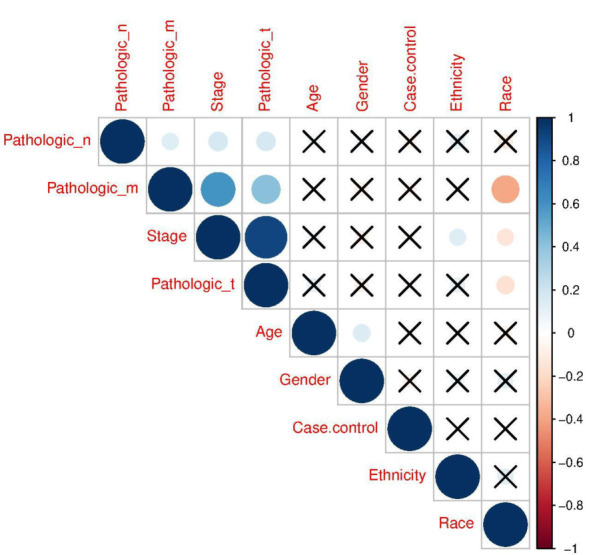
Correlation matrix shows significant co-relationship between clinical and demographic influence variables in PRCC; blue and red circles are displayed as positive and negative correlations, respectively. The size of circle and color intensity are associated with the correlation coefficients. The values of correlation coefficients are presented at the color intensity bar and the nonsignificant correlation is displayed in crosses.

As seen in Figure 4, the relationship between candidate genes and clinical information has been investigated which shows a direct relationship between stage and tumor invasion (pathologic T) and metastasis (pathologic M); tumor invasion and metastasis also show a significant relationship with each other. A correlation of less than 0.3 is considered weak, between 0.3 and 0.6 moderate, and more than 0.6 strong.

### ROC curve for identification of diagnosis markers

**Figure 5:**
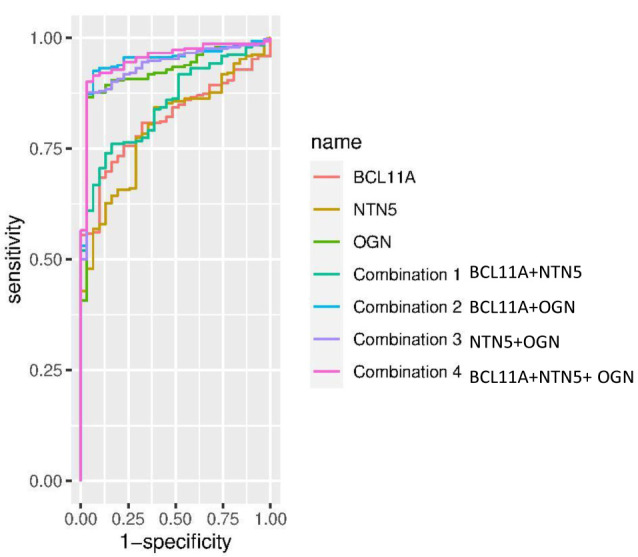
Combine ROC curve of BCL11A, NTN5, and OGN genes (combination 1 = BCL11A + NTN5, combination 2 = BCL11A + OGN, combination 3 = NTN5 + OGN, combination 4 = BCL11A + NTN5 + OGN)

Our results showed that among the three genes in question, OGN had the highest specificity and sensitivity (0.968 and 0.866, respectively); also, the combination of BCL11A-NTN5-OGN genes with specificity and sensitivity of 0.968 and 0.901, respectively, can be used as a diagnostic biomarker for PRCC (Figure 5, Table 4).

**Table 4:** Results of diagnosis tests performed for *BCL11A, NTN5, OGN*, and their combinations.

Diagnosis biomarker and their combinations	AUC	SE	SP	Cutoff	ACC	TN	TP	FN	FP	NPV	PPV	Coefficients	Degrees of Freedom	Null Deviance	AIC
BCL11A	0.815	0.685	0.903	0.928	0.706	28	200	92	3	0.233	0.985	0.8090	321	204.2	184.4
NTN5	0.798	0.568	0.935	0.930	0.604	29	166	126	2	0.187	0.988	1.27660	321	204.2	178.2
OGN	0.921	0.866	0.968	0.840	0.876	30	253	39	1	0.435	0.996	-1.042	321	204.2	127.7
Combination 1 (BCL11A-NTN5)	0.849	0.740	0.871	0.9	0.752	27	216	76	4	0.262	0.982	1.1148	320	204.2	167.6
Combination 2 (BCL11A-OGN)	0.946	0.925	0.935	0.799	0.926	29	270	22	2	0.569	0.993	-1.0673	320	204.2	113.6
Combination 3 (NTN5-OGN)	0.934	0.877	0.968	0.861	0.885	30	256	36	1	0.455	0.996	-0.9452	320	204.2	120.8
Combination 4 (BCL11A-NTN5-OGN)	0.951	0.901	0.968	0.849	0.907	30	263	29	1	0.508	0.996	-0.9946	319	204.2	112.4

### Validation

As a result of the GEO analysis of the datasets, it was found that OGN and BCL genes were identified in 40% of the datasets and NTN genes in 20% of the datasets.

## Discussion

An estimated 10–20% of all renal cell cancers are characterized by PRCC, which is the second most common histological type of RCC. Rapid progress made in explaining the molecular basis of this type of neoplasm in recent years has been remarkable; however, it is still not possible to provide a reliable molecular biomarker for detecting PRCC presence and grade of malignancy in daily clinical practice (27). In advanced stages, late diagnosis of this cancer has resulted in treatment failures and reduced survival rates (28). It is becoming increasingly important to find diagnostic biomarkers that can be used to detect early stage cancer and prevent it from progressing. This study aimed to develop diagnostic biomarkers for PRCC which can be used either alone or combined with other diagnostic biomarkers. Based on the results of the analysis listed in Table 3, 10 genes were selected for further analysis, 6 of which showed upregulation and 4 showed downregulation; then, ROC curves were drawn for three genes—BCL11A, NTN5, and OGN.

Reporting the role of BCL11A (B-cell lymphoma/leukemia 11A) in malignant solid tumors is rare, but overexpression of BCL11A has been detected in some malignant solid tumors, suggesting that this gene may be a valuable diagnostic and prognostic tool for these tumors (29). In LSCC (laryngeal squamous cell carcinoma) tissues, high levels of BCL11A were found and correlated with advanced lymphatic metastasis stages with poor prognoses. It has been shown that overexpression of BCL11A increases LSCC proliferation in vitro and in vivo; however, overexpression also causes high levels of MDM2 expression in LSCC cells, which interferes with the activity of p53 (30). Amplification of BCL11A has also been demonstrated in lung squamous cell cancers (SCC), with the highest concentration of amplification found in samples from NSCLC (non-small cell lung cancer) without metastases. The expression of BCL11A was greater in patients with early stage cancer, suggesting that the activation of BCL11A proto-oncogene may occur at an early stage in lung cancer. Thus, BCL11A may play a role in diagnosing and predicting the prognosis of patients with lung cancer, particularly those with early stage lung squamous carcinoma (31, 32). According to research, BCL11A expression levels decreased with increasing histological malignancy in breast cancer cases as well as cell lines. It was also negatively correlated with the size of primary tumors. The expression of BCL11A in BC that did not express estrogen or progesterone receptors as well as triple-negative cases was significantly lower. Therefore, it is likely that BCL11A is downregulated during the process of cancer occurrence (33). Additionally, in another study, BCL11A and SOX11 proteins were observed to have a significant positive correlation in the blood, suggesting that the two proteins may be regulated by the same pathway (34). A group of genes known as SOX have been implicated in the development of the kidney. In the early kidney anlagen, SOX11 is expressed at a level of both mesenchymal and epithelial expression. SOX11 directly binds and regulates a locus control region of the protocadherin B cluster on a molecular level. SOX11 is restricted to the intermediate segment of the developing nephron, as it is necessary for the elongation of Henle’s loop during the later stages of kidney development (35, 36). A number of other members of the SOX family, such as SOX6, SOX12, and SOX2, have also been shown to be associated with clinicopathological features, which may provide an advantageous prognostic biomarker for ccRCC patients (37, 38). As with many genes, BCL11A is regulated in part by miRNAs, and the let-7 family of miRNAs seems to affect BCL11A expression (39). Let-7 miRNAs play an important role in cell cycle control, differentiation, and apoptosis, and are widely considered tumor suppressors (40). Let-7 family members were found in abundance in urine cell-free supernatants of patients with ccRCC; let-7a outperformed the other miRNAs and may be a promising noninvasive biomarker for the detection of ccRCC (41).

Netrins are a family of highly conserved proteins that, in conjunction with semaphorins, slits, and ephrins, serve as neuronal guidance cues (42). In the beginning, these substances were known to play an important role in the development of the central nervous system, but over the last decade, they have been shown to participate in many other processes beyond the central nervous system development, including a pivotal role in the development of cancer (43). There is a correlation between mutations of members of the netrin family and cancer genetic characteristics, which suggests that these mutations may serve as potential biomarkers for prognosis and diagnosis. According to the studies, tumor mutations in members of the netrin family show unique distribution patterns correlated with cancer type, protein structure, and ethnicity (44). A study conducted in individuals with inflammatory bowel disease demonstrated that NTN1 is upregulated in fibroblasts associated with colorectal cancer promoting cancer cell stemness, thereby enhancing cancer cell progression (45, 46). Moreover, NTN1 has also been associated with the occurrence, development, survival, and clinical parameters of kidney cancer and non-small cell lung cancer (44, 47, 48). A number of clinical parameters, such as survival rates, are associated with the expression or methylation of NTNG1 and NTNG2. Besides, netrins are also altered by epigenetic and transcriptional factors in pan-cancer, which are associated with the activation of the EMT (epithelial–mesenchymal transition) pathway (44). Furthermore, a study found that NTN1/3/4/G1 were significantly downregulated and NTN5/G2 were significantly upregulated in ccRCC tissues compared to normal renal tissues, suggesting that netrin family members may be promising biomarkers for the detection of ccRCC (49). Among the netrins, NTN-5 (NTN5) has been the most recently discovered, receiving little attention thus far. Expressed in neuroproliferative zones, it is related to migration pathways in the adult brain (50). For the first time in this study, an increase in NTN5 expression was detected in PRCC, and based on the ROC curve, it can serve as a diagnostic biomarker for the diagnosis of this disease.

A wide range of cells secrete small leucine-rich proteoglycans (SLRPs), which are involved in a variety of processes. These processes include protein-protein interactions, signal transduction, cell adhesion, and DNA repair (51). In addition to their ability to bind collagen, the SLRP family also performs outside-in signaling (52). In addition to being one of the SLRPs, osteoglycin (OGN) is a member of the family of extracellular proteoglycans, which have several leucine-rich repeats, just like other members of the family. In addition to binding collagen and several growth factors, OGN may also be involved in remodeling the extracellular matrix (ECM); EGFRs (epidermal growth factor) and IGFs (insulin growth factor) are among the receptors (53). Many cancer cell lines lack the expression of OGN, suggesting that it may serve as a tumor suppressor gene in the development of cancer (54). Both ECRG4 and OGN function as tumor suppressors in the bladder, with ECRG4 overexpression inhibiting NF-kB signaling and promoting NFIC/OGN signaling in bladder cancer cells (55). OGN expression is associated with increased survival and decreased recurrence of colorectal cancer. OGN also suppresses the EGFR/AKT/Zeb-1 axis, reversing the EMT (56). However, elevated expression of OGN is associated with the EMT process and shorter overall survival in ovarian carcinoma tissues (57). In another study in breast cancer, OGN levels were significantly reduced in breast cancer tissue; overexpression of OGN significantly inhibited cell proliferation, migration, and invasion and reversed EMT phenotypic changes. Furthermore, OGN’s tumor suppressor activity in BC is demonstrated to be mediated by its effect on the PI3K/AKT/mTOR pathways (58). A significant reduction in OGN expression was observed in gastric cancer tissues, and a decrease in OGN expression was associated with more lymph node metastasis and poor differentiation status, both indications that a cancer has advanced. These results suggest that OGN downregulation might contribute to the progression of gastric cancer and could be utilized for the diagnosis and monitoring of cancer (53). A study investigated proteomics for diagnostic biomarkers of laryngeal cancer, and four differential proteins (PFN1, NCL, CNDP2, and OGN) with expressional changes were selected to test for differential expressions. Sone of the four proteins were shown to be potential biomarkers for detection or therapeutic targets of human laryngeal carcinoma (59). As a result, based on the role of OGN in cancer and its potential as a biomarker, in this study, the role of this gene in PRCC has been shown for the first time, and based on the ROC curve, it is possible for it to be used alone or in combination with two other genes—BCL11A and NTN5—to detect this disease.

## Data Availability

Data and material would be access by request to the corresponding authors.
